# Distinct Distribution Pattern of Hepatitis B Virus Genotype C and D in Liver Tissue and Serum of Dual Genotype Infected Liver Cirrhosis and Hepatocellular Carcinoma Patients

**DOI:** 10.1371/journal.pone.0102573

**Published:** 2014-07-17

**Authors:** Somenath Datta, Shrabasti Roychoudhury, Alip Ghosh, Debanjali Dasgupta, Amit Ghosh, Bidhan Chakraborty, Sukanta Roy, Subash Gupta, Amal Kumar Santra, Simanti Datta, Kausik Das, Gopal Krishna Dhali, Abhijit Chowdhury, Soma Banerjee

**Affiliations:** 1 Centre for Liver Research, School of Digestive and Liver Diseases, Institute of Post Graduate Medical Education and Research, Kolkata, India; 2 Department of Gastro-Intestinal surgery, School of Digestive and Liver Diseases, Institute of Post Graduate Medical Education and Research, Kolkata, India; 3 Centre for Liver and Biliary Surgery, Indraprastha Apollo Hospital, New Delhi, India; 4 Department of Hepatology, School of Digestive and Liver Diseases, Institute of Post Graduate Medical Education and Research, Kolkata, India; 5 Department Gastroenterology, School of Digestive and Liver Diseases, Institute of Post Graduate Medical Education and Research, Kolkata, India; Saint Louis University, United States of America

## Abstract

**Aims:**

The impact of co-infection of several hepatitis B virus (HBV) genotypes on the clinical outcome remains controversial. This study has for the first time investigated the distribution of HBV genotypes in the serum and in the intrahepatic tissue of liver cirrhotic (LC) and hepatocellular carcinoma (HCC) patients from India. In addition, the genotype-genotype interplay and plausible mechanism of development of HCC has also been explored.

**Methods:**

The assessment of HBV genotypes was performed by nested PCR using either surface or HBx specific primers from both the circulating virus in the serum and replicative virus that includes covalently closed circular DNA (cccDNA) and relaxed circular DNA (rcDNA) of HBV from the intrahepatic tissue. The integrated virus within the host chromosome was genotyped by Alu-PCR method. Each PCR products were cloned and sequences of five randomly selected clones were subsequently analysed.

**Results:**

HBV/genotype D was detected in the serum of all LC and HCC patients whereas the sequences of the replicative HBV DNA (cccDNA and rcDNA) from the intrahepatic tissue of the same patients revealed the presence of both HBV/genotype C and D. The sequences of the integrated viruses exhibited the solo presence of HBV/genotype C in the majority of LC and HCC tissues while both HBV/genotype C and D clones were found in few patients in which HBV/genotype C was predominated. Moreover, compared to HBV/genotype D, genotype C had higher propensity to generate double strand breaks, ER stress and reactive oxygen species and it had also showed higher cellular homologous-recombination efficiency that engendered more chromosomal rearrangements, which ultimately led to development of HCC.

**Conclusions:**

Our study highlights the necessity of routine analysis of HBV genotype from the liver tissue of each chronic HBV infected patient in clinical practice to understand the disease prognosis and also to select therapeutic strategy.

## Introduction

Persistent infection with non-cytocidal hepatitis B virus (HBV) is the leading cause of chronic hepatitis, liver cirrhosis (LC) and hepatocellular carcinoma (HCC). The covalently closed circular DNA (cccDNA), relaxed circular DNA (rcDNA) and the replicative RNA intermediate of HBV, the pregenomic RNA (pgRNA) in the infected hepatocyte are accountable for the persistence of chronic HBV infection. The cccDNA serves as the repository of HBV DNA throughout the natural history of chronic hepatitis B (CHB), even after sero-clearance of hepatitis B surface antigen (HBsAg) when HBV persists at a low replicative and transcriptional level [1] instigating liver damage slowly. Integration of HBV in the genome of the host hepatocyte has also been reported as frequent event in the due course of persistent viral infection [2], which is one of the causes of the development of HCC [3], [4].

Beyond the complex host-viral immune interplay, viral factors such as the genotypes of HBV have profound impact on the clinical outcome of HBV infection [5]. Systematic sequencing of complete HBV genome has identified ten different HBV genotype variants (A–J) that differ by their degree of pathogenicity and response towards therapy [6]–[8]. These genotype variants have interesting geographical and ethnic distribution pattern across the globe [9]. Genotype B and C are confined in most endemic countries where vertical or perinatal transmission is the route of viral spread [10], [11], whereas genotype A, D, E, F and G are predominating in those area where horizontal transmission is the route of viral transmission [12]. In India, genotype A and D are mostly circulating among different states [13] and genotype C has been reported exclusively from East (Kolkata) [14]–[16], Northeast (Arunachal Pradesh) [17] and Eastern part of North India (Uttar Pradesh) [18]. All the single HBV-genotype infection has the propensity to develop liver diseases but in an HBV endemic area, co-infection or super infection with multiple HBV genotype variants are often observed [19], [20]. The importance of mixtures of genotypes infection in the pathogenicity of HBV carriers especially the presence of minor viral quasi-species had rarely been studied [21]–[23]. Furthermore, CHB patients infected with mixtures of genotypes showed poor response to interferon therapy and emerged with higher number of classical precore or basal core promoter mutations associated with the increased viral replication levels [24]. Thus complex interplay between genotypes might regulate the degree of chronicity of infection and response towards therapy. However, a very little information about genotypic co-infection has been documented from India. Two epidemiological studies have reported about 6% and 7.6% of mixed HBV infection with genotype A and D from serum sample of HCC patients [25], [26].

The present study has assessed the distribution of HBV genotypes in the serum for circulating virus (CV) and in the intrahepatic tissue for the replicative virus (RV) (cccDNA/rcDNA) and integrated virus (IV) in HBsAg positive LC and HCC patients. This study has also investigated the interplay of viral genotypes in the disease progression with two HBV genotypes C and D, which are mostly associated with the advanced form of liver diseases such as LC and HCC.

## Materials and Methods

### Study subjects

Twelve HBV positive HCC and eight LC patients were included in the study who were undergoing orthotropic liver transplantation at (i) Centre for Liver and Biliary Sciences, Indraprastha Apollo Hospital, Delhi (n = 16) and (ii) School of Digestive and Liver diseases, Institute of Post Graduate Medical Education and Research, Kolkata (n = 4) in between February 2010 to March 2013. Patients were sero-negative for HIV, HCV and with no history of chronic alcohol intake.

Liver cirrhosis (LC) (n = 8) was diagnosed based on histological feature of cirrhosis in explanted tissues supplemented with clinical, radiological and endoscopic data. HCC (n = 12) was diagnosed also by histological topographies supplemented with radiological analysis through spiral/triphasic dynamic CT scans and elevated alpha-feto protein level. All HCC patients had underlying cirrhosis.

Blood was collected before surgery from every patients and serum was separated and preserved at −80°C. Tissues were collected in RNA later (Ambion, USA), equilibrated at 4°C overnight and then stored at −80°C. In HCC cases, both tumour and adjacent cirrhotic non-tumour tissues were collected.

### Ethics statement

The Institutional ethical review committee has approved the study. All patients from whom blood and tissue were collected provided written informed consent.

### Serological parameters test

The presence of HBsAg, HBeAg, anti-HBeAg, anti-HCV was tested using commercially available ELISA kit from General Biologicals, Taiwan and Biomerieux, Boxtel, Netherlands. Each sample was subjected to blood biochemistry such as ALT (SGPT), AST (SGOT), total protein, albumin, globulin, bilirubin, prothrombin time (INR) using commercially available kits from Bayer Diagnostics, India.

### Extraction of chromosomal and viral DNA from tissue and serum samples

Genomic DNA was extracted from frozen tissues by proteinase K digestion followed by phenol/chloroform/isoamyl alcohol extraction [27] and DNA was precipitated with alcohol at −80°C for overnight to collect small viral DNA along with chromosomal DNA.

HBV DNA was extracted from 200 µL of serum using the QIAamp DNA Mini Kit following manufacturer protocol (Qiagen, Valencia, CA, USA).

### Detection of integrated viral (IV) DNA by Alu-PCR method

Alu-specific PCR was used to detect the IV DNA in human chromosome [28]. In brief, Alu-specific PCR was performed using several combinations of primer pairs from HBV sequences and human Alu repeat sequence (Table 1). To avoid the undesirable amplifications between Alu sequences, initial 10 cycles were performed using primers with dUTPs which were subsequently degraded by uracil DNA glycosylase treatment and then amplified with the specific primers (without dUTPs) from HBV sequence and the universal tag sequence present in the Alu-specific primer.

**Table 1:** List of primers used to generate PCR products and promoter construct.

Primer Name	Nucleotide sequences of primers	Nucleotide position in HBV
**Alu-PCR**		
A3 (Alu3)	AGUGCCAAGUGUUUGCUGACGACUGCACUCCAGCCUGGGCGAC	–
A5 (Alu5)	CAGUGCCAAGUGUUUGCUGACGCCAAAGUGCUGGGAUUACAG	–
Tag3	CAAGTGTTTGCTGACGACTGCA	–
Tag5	CAAGTGTTTGCTGACGCCAAAG	–
uPx1	ACAUGAACCUUUACCCCGUUGC	1131–1152
Px2	TGCCAAGTGTTTGCTGACGC	1174–1193
Px3	CTGCCGATCCATACTGCGGAAC	1258–1279
uPs1	ACACGGCGGUAUUUUGGGGTGGAG	3042–3065
ps2	CAGGCTCAGGGCATATTGACAA	3070–3091
ps3	TCCTGCTGGTGGCTCCAGTTC	55–75
**HBX Region**		
F6	TGCCGATCCATACTGCGGAA	1259–1278
HBX-1296F	CAAGCTTGCTCGCAGCCGGTCTGGAGC	1296–1316
R6	ACAGCTTGGAGGCTTGAACA	1861–1880
HBX-1833R	GGCGGCCGCGGCAGAGGTGAAAAAGTTG	1817–1835
**Surface Region**		
F3	CGCCTCATTTTGTGGGTCAC	2801–2820
F10	GACCACCAAATGCCCCTATC	2298–2317
R5	AAAGCCCAAAAGACCCACAAT	997–1017
R8	CTTTGACAAACTTTCCAATCAAT	973–995
**GRP78 promoter**		
GRP78 F	GAAGCTTCAATAGGTCAATCTGTCTGTGCTG	
GRP78R	GCTCGAGGAAGGGAGAACAAGCAGTAGAGAA	

PCR products were cloned (CloneJet PCR cloning Kit, Fermentas, USA) and then five individual clones were sequenced using BigDye terminator v 3.1 cycle sequencing kit (Applied Biosystems, USA) on an automated DNA sequencer (ABI Prism 3130) and sequences were analyzed using NCBI-Blast tool to detect IV sequences.

### Amplification of viral genes from serum and tissue

HBx and HBV surface regions were amplified by nested PCR using HBV DNA isolated from serum to determine the sequences of CV and chromosomal DNA isolated from liver tissue was used to amplify replicative viral DNA (RV) that includes cccDNA/rcDNA of HBV (Table 1). The PCR products were cloned (CloneJet PCR cloning Kit, Fermentas, USA) and five clones of each product were sequenced.

### Sequence alignment and genotype detection

Viral genotype was ascertained by comparing the nucleotide sequences of HBs region of HBV from 195 clones (13 samples ×3 forms of HBV DNA ×5 clones) along with the direct sequences of the PCR product to the 55 reference sequences representing each of the ten HBV genotypes (A–J) retrieved from the Genbank using ClastalW (MEGA 4.1) sequence alignment tool. In few of the samples the full-length HBx was considered for genotyping of all three forms of the viruses [14] in which surface region was not detected by Alu-PCR method.

### Cell culture and Transfection

HepG2 and Huh7 cell lines were maintained in Dulbecco’s modified Eagle’s medium (DMEM) containing 10% fetal bovine serum (Invitrogen, CA, USA) and 1% penicillin-streptomycin (Sigma-Aldrich, MO, USA) under 5% CO2 at 37°C. Plasmids were transfected using either Lipofectamine 2000 (Invitrogen, CA, USA) or Xtremegene (Roche, Basel, Switzerland) following the manufacturers’ protocols. Each experiment was repeated three times.

### Quantification of double strand breaks (DSBs) and reactive oxygen species (ROS) production

To quantify the amount of DSBs produced in presence of HBV, 4×10^4^ Huh7 cells were grown on each well of 4 well chamber slides, transfected with 300 ng of plasmids, pTriEX-HBV/D and pTriEX-HBV/C carrying 1.2× HBV/genotype D and genotype C genome (kindly gifted by Prof. Fabien Zoulim, INSERM, France to Dr. S. Datta) respectively and pTriEX control vector separately. After 72 hours of transfection, cells were fixed, immuno-stained with rabbit polyclonal anti-γ-H2AX antibody (Ab11174, Abcam) followed by counter-stained with goat anti-rabbit IgG-FITC (Molecular probe, Life Technologies) secondary antibody and observed under confocal microscope after mounting with ProLong Gold anti-fade reagent with DAPI (Molecular Probe, Invitrogen). Number of foci in each cell per field was counted and average of ten fields was plotted.

To quantify ROS, after transfection following the above protocol, cells were incubated with 2′,7′–dichlorofluoresceindiacetate (DCFDA) for half an hour and then either analyzed on FACS caliber (BD Biosciences, CA, USA) after trypsinization and fluorescent intensity (mean +/− standard deviation) was plotted or cells were fixed and observed under confocal microscope.

### Luciferase assay

The full length promoter of GRP78 was amplified from HepG2 chromosomal DNA using two primers GRP78F/GRP78R, where XhoI and HindIII sites were appended for efficient cloning (Table 1) in pGL3-Basic vector (kindly gifted by Dr. Thomas Schmittgen, OSU, USA). The sequence of the promoter was further confirmed by sequencing using vector specific primers. The pGL3-GRP78 promoter construct (100 ng) was then co-transfected with 500 ng of each plasmid pTriEX vector, pTriEX-HBV/D and pTriEX-HBV/C separately in 1×10^5^ of HepG2 cells seeded on 12 well plates in triplicate. After 48 hours, luciferase activities were determined using the Dual-Luciferase Reporter Assay kit (Promega, Madison, USA) in luminometer. This experiment was also repeated in Huh7 Cell line. *p*<0.05 was considered as significant variation.

### Homologous recombination (HR) assay

The pDR-GFP reporter construct (Addgene, Cambridge, MA) was stably transfected in HepG2 cells and 5×10^5^ cells were plated on 6 well plate and then transfected with 3 µg of each plasmid, pTriEX-HBV/D, pTriEX-HBV/C and control vector separately. At 48 hour, 1 µg of pCBASceI (Addgene, Cambridge, MA) plasmid, which encoded I-SceI enzyme was transfected to induce a DSB in the construct and after 72 hours, cells were collected and the frequency of I-SceI break induced HR was measured by acquiring GFP positive cells in flow cytometer (BD Biosciences, CA, USA) [29].

### Statistical Analysis

GraphPad Prism 5 (GraphPad Software, La Jolla, California, USA) was used for statistical analysis. The independent Student t-test was used to compare data between two different groups. All the experiments were performed at least three times and mean value with standard deviation was presented. p<0.05 was considered as statistically significant.

### Accession numbers of submitted Nucleotide sequences

The nucleotide sequences from surface and HBx region of HBV isolates used for analysis in this paper are available in Genbank database http://www.ncbi.nlm.nih.gov/GenBank/index.html by the accession numbers KJ997762–KJ997911.

## Results

### Baseline and clinical characteristics of the study subjects

Twelve HCC with underlying cirrhosis and eight LC patients were included in the study after clinical, biochemical and histological confirmation of each subject (Table 2). HBV infection was authenticated by the presence of HBsAg in serum of all samples. All patients were HBeAg negative and anti-HBe positive.

**Table 2:** Baseline characteristics and biochemical parameters of LC and HCC patients.

Baseline Characteristics	LC, n = 8	HCC, n = 12
Gender (M:F)	8∶0	12∶0
Age [Years], mean±SD	42.6±13.2	55.2±7.05
HBeAg negativity	8	12
Anti-HBeAg positivity	8	12
AFP [IU/mL], median [range]	290 [33–482]	1136 [574–2326]
Albumin [g/dL], mean±SD	3.01±0.50	3.05±0.68
Total Bilirubin, [mg/dL], median [range]	5.55 [3.1–12.7]	3.2 [0.3–50.6]

### Distribution pattern of HBV genotypes in the serum and liver tissue of HCC and LC patients

To delineate the effect of genotype of HBV on the liver disease progression to HCC, 12 HCC and adjacent HCC and 8 LC tissues were subjected to further analysis. HBV genotype was detected from either surface (HBs) or HBx sequence and compared with the sequences of 10 HBV genotypes (A–J) retrieved from the database. HBV gene specific primers (Table 1) were used to amplify the circulating virus (CV) present in the serum. The same primer pairs could amplify genes from the replicative virus (RV) using both cccDNA and rcDNA of HBV as template from the intrahepatic tissue. The integrated virus (IV) was detected by Alu-PCR method [28] using one primer from viral gene and another from human Alu repeat region (Table 1). The assessment of HBV genotypes in all three forms of viruses: CV, RV and IV were possible from 13 (7/12 HCC and 6/8 LC) subjects, which were considered for subsequent analysis.

**Figure 1:**
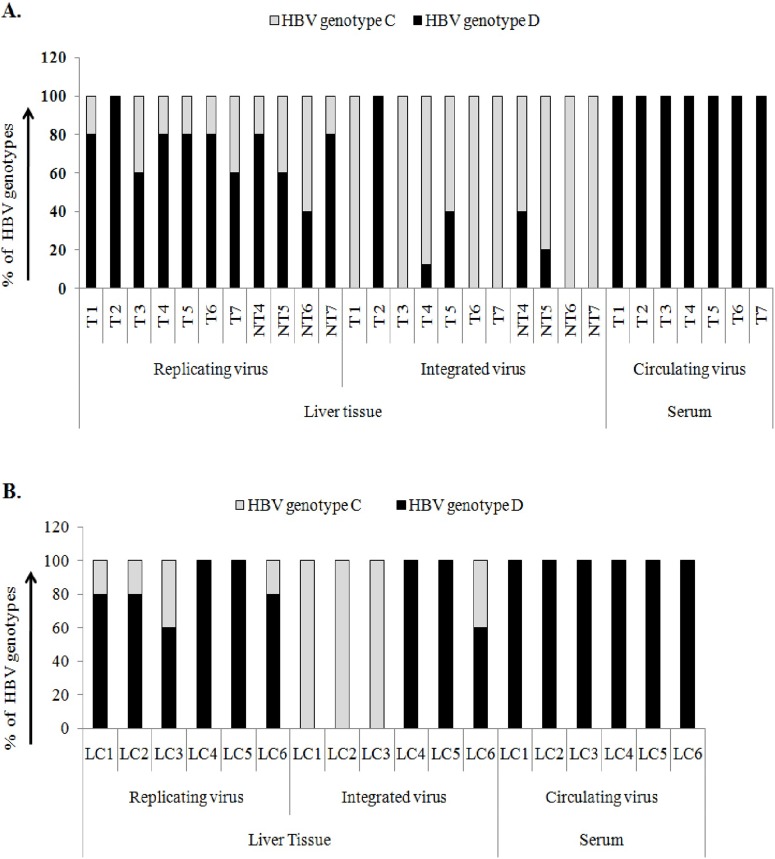
Distribution pattern of HBV/genotype C and HBV/genotype D in hepatocellular carcinoma (HCC) (A) and in liver cirrhotic (LC) (B) patients. HBV genotypes were detected from sequence analysis of five clones of amplified HBx or HBs region from chromosomal DNA and serum viral DNA using virus specific primer pairs. This will amplify replicating virus in liver tissue and circulating virus in serum. Genotypes of Integrated viruses were determined from sequence analysis of five clones of Alu-PCR product. T = tumor, NT = adjacent non-tumor and LC = liver cirrhosis.

Randomly selected 5 clones from each of the PCR product of 13 samples (total 195 clones) were subjected to sequence analysis. HBV/genotype D was confirmed from the sequence analysis of CV clones of 13 patients (7 HCC and 6 LC) (Figure 1A and 1B) and also from the direct sequencing of the amplified product, whereas the sequences of RV clones from intrahepatic tissues showed HBV/genotype C and D co-infection in liver tissues of 10 patients (10/13, 76.9%) distributed as 6/7 of HCC (85.7%) (Figure 1A) and 4/6 (66.6%) of LC tissue samples (Figure 1B) and remaining 3 patients showed only HBV/genotype D infection as detected in their serum. The sequence analysis of IV clones from 10 patients revealed the presence of only genotype C virion in 7 patients (7/10, 70%) and both HBV/genotype C and D in remaining 3 patients (3/10, 30%) (Figure 1A and 1B). It was also noted that genotype C containing clones were over-represented among the IV clones but sequences of RV clones showed more genotype D clones (Figure 1A and 1B) indicating present infection. The distribution of HBV genotypes in non-HCC liver tissues was similar to HCC tissues (Figure 1A).

The mechanism of the preferential integration of HBV/genotype C in the host chromosomes compared to HBV/genotype D was further investigated, as integration of HBV genome in host chromosome is one of the causes of progression of chronic liver diseases to HCC.

### HBV/genotype C generated more double strand breaks (DSBs) than genotype D during the course of infection

**Figure 2:**
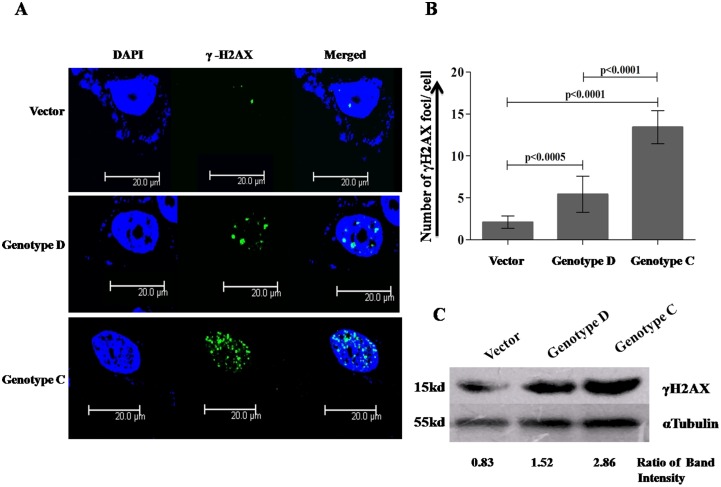
Quantification of DNA double strand breaks generated after HBV infection. Huh7 cells (4×10^4^) were transfected with control pTriEX vector and pTriEX-HBV/D, pTriEX-HBV/C plasmids, which contain 1.2× HBV genome, separately in 4 wells glass chamber slide. After 48 hours, (A) cells were fixed, immuno-stained with γ-H2AX antibody and observed under confocal microscope. Scale bar corresponds to 20 µm. (B) The number of foci per cell in ten different fields was counted and average of foci number per cell was plotted. (C) Total protein was isolated and western blot analysis was performed with γ-H2AX antibody. The ratio of band intensity between γ-H2AX and tubulin was determined. The experiment was repeated three times. *p*<0.05 was considered as significant variation.

Previous studies had shown that DSBs serve as potential targets for HBV DNA integration [29]. To measure the frequency of DSBs generated after HBV infection with two genotypes, D and C, Huh7 cells were transfected with pTriEX-control vector, pTriEX-HBV/D and pTriEX-HBV/C separately and after 48 hours, cells were immuno-stained with γ-H2AX antibody as DSBs are always followed by phosphorylation of H2AX, a variant of the Histone 2A protein family by PI3 kinases Ataxia telangiectasia mutated (ATM) or ATM-Rad3 related (ATR) protein (Figure 2A). The average number of γ-H2AX foci per cell in presence of HBV/genotype C was significantly higher than HBV/genotype D (p<0.0001) (Figure 2A and 2B). The result was also confirmed by western blot analysis with pTriEX-HBV/D and pTriEX-HBV/C transfected cell lysates (Figure 2C).

### HBV/genotype C generated more reactive oxygen species (ROS) than HBV/genotype D infected cells

**Figure 3:**
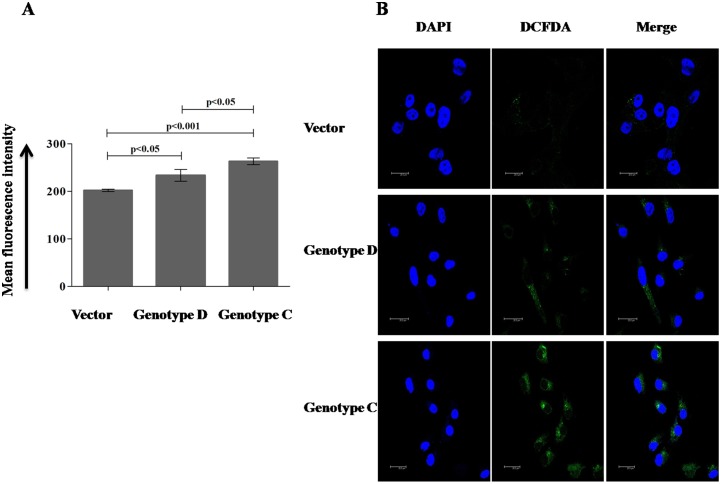
Quantification of ROS generated after HBV/genotype D and HBV/genotype C infection. Huh7 cells were transfected with control pTriEX vector and pTriEX-HBV/D and pTriEX-HBV/C plasmids, which contain 1.2× HBV genome independently. After 48 hours, cells were treated with DCFDA and DCF positive cells were quantified on (A) FACSCaliber and (B) Confocal microscope. Scale bar corresponds to 20 µm. *p*<0.05 was considered as significant.

It has been well accepted that generation of ROS can cause oxidative DNA damages, which acts as an important mutagenic and apparently carcinogenic factor for cancer development [30], [31] and HBx protein of HBV enhances the ROS production [32]. To elucidate the effect of HBV genotype on ROS production, Huh7 cell lines were again transfected following the above mentioned protocol and after 48 hours, the amount of ROS was quantified by using an oxidation sensitive fluorescence dye, 2′,7′-dichlorodihydrofluorescien diacetate (DCFDA) in Flow cytometer and also in confocal microscope (Figure 3A and 3B). The mean fluorescence intensity of DCF in presence of HBV/genotype C was significantly higher than HBV/genotype D (p<0.01) (Figure 3A) and the number of DCF positive cells observed in confocal microscope was also similar to the data of Flow cytometer (Figure 3B).

### Endoplasmic reticulum (ER) stress induced by HBV proteins of genotype C was more than genotype D

**Figure 4:**
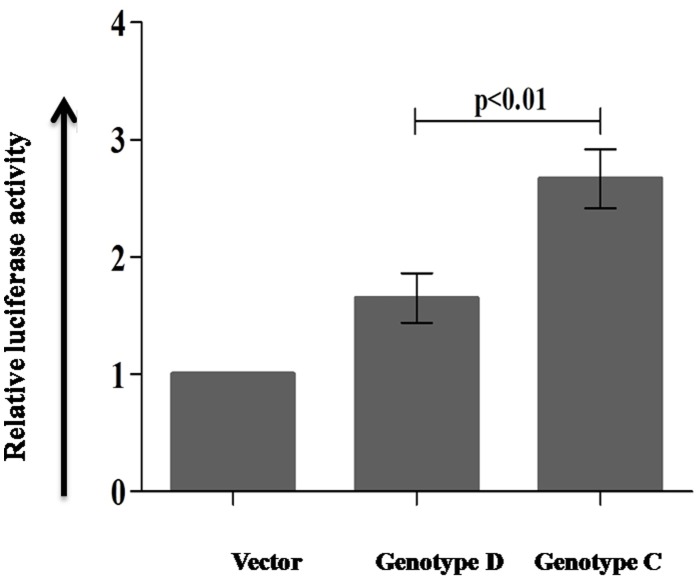
ER stress was detected in HBV infected cells. The pGL3-GRP78 promoter construct was co-transfected with control pTriEX vector and pTriEX-HBV/D and pTriEX-HBV/C plasmids, which contain 1.2× HBV genome, separately in HepG2 cell line. After 48 hours, luciferase activity was detected in luminometer. *p*<0.05 was considered as significant.

ER serves as an essential organelle in viral replication and maturation. Recent studies have suggested that viral proteins can induce ER stress by accumulating a large amount of unfolded and misfolded viral proteins in ER of infected cells [33]. Subsequently, this stress can influence the ROS production [34], [35] and induces stress signal chaperon protein Glucose regulated protein 78 (GRP78). This in turn generates DNA damages, deregulates signaling pathways and activates oncogenic transformation of cells. To quantify the ER stress produced by HBV/genotype D and genotype C, HepG2 cells were transfected with pTriEX control vector, pTriEX-HBV/D and pTriEX-HBV/C plasmids separately along with pGL3-GRP78 promoter-luciferase reporter construct and after 48 hours, luciferase activity was determined. The average luciferase activity in presence of HBV/genotype C was 1.6 fold higher than HBV/genotype D (p<0.01, Figure 4).

Thus, in comparison to the amount of ROS production in HBV/genotype D infection, infection with HBV/genotype C might generate more ROS by inducing ER stress, which led to the accumulation of larger number of DSBs in the host DNA.

### HBV/genotype C infected cells showed higher homologous recombination (HR) efficiency in comparison with the cells infected with HBV/genotype D

**Figure 5:**
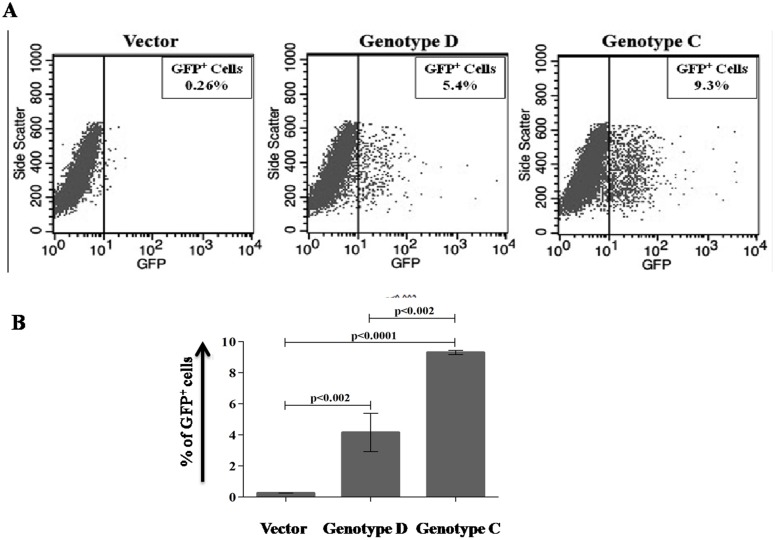
Intracellular homologous recombination (HR) frequency was detected in HBV/genotype D and genotype C infected cells. (A) The reporter-construct pDR-GFP containing stable HepG2 cell lines were transfected with control pTriEX vector and pTriEX-HBV/D and pTriEX-HBV/C plasmids, which contain 1.2× HBV genome separately. After 48hours, cells were transfected with ISceI producing plasmid pCBASceI and GFP positive cells were quantified on flow cytometer at 96 hours and (B) average HR frequency of three experiments was presented graphically. *p*<0.05 was considered as significant variation.

Although the mechanism of HBV integration in human genome is controversial, several studies have depicted that the virus might be integrated into the host genome by random recombination [36]–[38]. The efficiency of cellular homologous recombination in presence of HBV/genotype D and HBV/genotype C was studied in HepG2 cells stably transfected with a reporter construct pDR-GFP. After three days of transfection with plasmid pTriEX-HBV/D, pTriEX-HBV/C and control vector separately, a single DSB was introduced within the construct by expressing the I-SceI endonuclease from a plasmid, pCBASceI. After 2 days of transfection, recombination efficiency was determined by comparing GFP positive cells in flow cytometer. A significantly higher number of GFP-positive cells were detected in the presence of HBV/genotype C than in the presence of HBV/genotype D (Figure 5), implying higher recombination potential of HBV/genotype C infected host cells.

Thus high number of DSBs generated from oxidative stress might initiate random recombination and subsequently, it has contributed to the formation of an unstable environment that facilitated malignant transformation of hepatocyte.

## Discussion

We have demonstrated that the co-infection with different HBV genotypes is a frequent event in hepatocellular carcinoma (HCC) and also in premalignant liver cirrhosis (LC) stage of liver diseases in the geographical area where multiple genotypes of HBV co-exist, depicting the synergistic effect of HBV genotypes on the development of LC and HCC. This is the first delineation exploring the distribution of HBV genotypes in liver tissue and serum of the same patient. The fascinating observation was the detection of the single HBV genotype in the serum of the LC and HCC patients whereas the intrahepatic tissue of the same patient showed the existence of mixtures of HBV genotypes after sequence analysis of five randomly selected clones from the amplified product of either “a” determinant region of the surface [39] or HBx gene [14].

As viral genotypes are the potent viral factor that influences the liver disease progression and determine the outcome of anti-viral therapy in chronic HBV infected patients [40], the co-infection with mixed HBV genotypes creates a more complex environment to comprehend the disease progression.

The patients included in this study were HBeAg negative and anti-HBeAg positive and had no declaration about HBV genotype at the early onset of the disease. A radical shift in distribution of HBV genotype in both LC and HCC patients indicated either co-existing of HBV/genotype D as minor pool with genotype C or HBV/genotype D might super-infected in a patient where HBV/genotype C was pre-existed. In accordance with the first hypothesis, one German study also showed genotype shifting (27.2%, 6/22) after HBeAg sero-conversion in children but mixed genotype was not detected before sero-conversion [41] indicating the possibility of taking over of the fittest minor strain, when viremia was dropped. While in the later case, the probability of super-infection might be difficult to establish in a patient where immunity against one HBV genotype is already present throughout the liver. In our study, the co-infection with HBV genotype C and D was confirmed from the sequence analysis of HBV surface or HBx clones from intrahepatic tissues of 10 patients, whereas serum of the same patients showed infection with only HBV/genotype D. Again, the sequence analysis of the integrated viruses in 7 patients (70%, 7/10) had confirmed the presence of only HBV/genotype C, whereas the remaining three patients showed both genotype C and D clones but overall genotype C clones were overrepresented (Figure 1A and 1B). Integration of HBV genome into host chromosome is frequently observed in different stages of chronic HBV infection including LC and HCC [2]–[4] and it is considered as one of the mechanisms of generating chromosomal rearrangements which eventually lead to development of HCC [42]. So our results might be relevant to the context that HBV/genotype C infection which has been considered as more aggressive strain in expanding liver diseases [43] might be accountable for more favorable prognosis of the liver diseases to the development of LC and HCC [44] and HBV/genotype D had emerged lately after sero-conversion.

Again, several evidence-based studies had demonstrated that HBV/genotype C infection is more in HBeAg positive patients and the mean age at sero-conversion is quite late, such as 40±10 years, indicating longer duration of active replication generating more hepatocyte damages [45] whereas HBV/genotype D was reported in younger patients less than 40 years [25]. The average age of the patients included in our study were 42±13 years in LC and 55±7 years in HCC (Table 2), all patients were sero-converted and HCC patients had underlying cirrhosis. This result was again in accordance with the hypothesis raised by Gerner *et* *al*, that after sero-conversion the minor strain of HBV/genotype D might have emerged [41].

Although, a detailed follow-up study from early stage of HBV infection would help us to understand the mechanism of genotype switch in co-infection, an alternative consequences could be that mutations acquired in HBV/genotype D genome during its due course of infection might alter the immune epitopes, leading to escape from immune selection pressure, whereas the other genotype may lead to development of immune response which causes liver damage [24]. Mutation analysis in a comparatively larger cohort might strengthen this hypothesis and we are planning that.

Moreover, to support our hypothesis, we have also showed that HBV/genotype C could generate more ROS (Figure 3A and 3B) and DNA double strand breaks (DSBs) (Figure 2A and 2B) than HBV/genotype D in hepatoblastoma cell line, Huh7 and hepatocellular carcinoma cell line HepG2 (data not shown) inferring the probability of integration of HBV/genotype C in host chromosome is more than HBV/genotype D. In 85–90% of HCC cases, insertion of HBV genome in host chromosome has been observed and the recent genome wide sequencing studies have confirmed that these integration events are at random [46], [47]. After DNA damages, homologous recombination (HR) and non-homologous end joining (NHEJ) are the preferred recombination pathways to contend the damages whereas misrepair of DSBs lead to chromosomal rearrangements [48]. As observed in our study, the HBV/genotype C infection generated larger number of DSBs in host chromosomes and produced higher rate of HR than the HBV/genotype D infection, suggesting HBV/genotype C might have higher risk of development of HCC in comparison to HBV/genotype D infection (Figure 4).

Therefore, despite the limitation in small number of samples, this data adds unique information to the available studies on the pathological relevance of HBV genotypes. The high frequency of genotypic co-infection in LC and HCC patients implicates that the screening of HBV from liver tissue should be compulsory in clinical practice for each CHB patient, especially, patient with sustained virological response to identify small HCC, so that curative therapy could be commenced earlier. Anti-viral therapy should be started at the very early stages of HBV infection when viral genome may not get the chance to integrate.
