# Correction: Distinct Distribution Pattern of Hepatitis B Virus Genotype C and D in Liver Tissue and Serum of Dual Genotype Infected Liver Cirrhosis and Hepatocellular Carcinoma Patients

**DOI:** 10.1371/journal.pone.0110932

**Published:** 2014-10-15

**Authors:** The PLOS ONE Staff

The sixth author's name is spelled incorrectly. The correct name is: Bidhan Chandra Chakraborty.

The seventh author's name is spelled incorrectly. The correct name is: Sukanta Ray.

The second sentence of the “Cell culture and Transfection” section of the Materials and Methods incorrectly references Xtremegene. It should read X-tremeGENE HP.

In the Acknowledgements, Bidhan Chandra Chakraborty should also be listed as having a registered PhD with the West Bengal University and health sciences.

There are errors in the Author Contributions. The correct contributions are: Conceived and designed the experiments: KD GKD AC SB. Performed the experiments: Somenath Datta S. Roychoudhury Alip Ghosh DD Amit Ghosh BCC AKS. Analyzed the data: Somenath Datta SB. Contributed reagents/materials/analysis tools: S. Ray SG Simanti Datta. Contributed to the writing of the manuscript: SB.
